# Cognitive deficits including executive functioning in relation to clinical parameters in paediatric MS patients

**DOI:** 10.1371/journal.pone.0194873

**Published:** 2018-03-22

**Authors:** Eva Wuerfel, Almuth Weddige, York Hagmayer, Rebecca Jacob, Lisa Wedekind, Wiebke Stark, Jutta Gärtner

**Affiliations:** 1 Department of Paediatrics and Adolescent Medicine, Division of Paediatric Neurology, University Medical Center Göttingen, Georg August University Göttingen, Göttingen, Germany; 2 Institute of Medical Genetics and Pathology, University Hospital Basel, Basel, Switzerland; 3 Human Genomics Research Group, Department of Biomedicine, University of Basel, Basel, Switzerland; 4 Department of Psychology, University of Göttingen, Göttingen, Germany; Nathan S Kline Institute, UNITED STATES

## Abstract

**Background:**

A number of studies have investigated cognitive impairment in paediatric patients with multiple sclerosis (MS) but deficits regarding executive functions have not been comprehensively assessed up to now. This study was meant to explore cognitive impairment in German paediatric MS patients with a focus on deficits in executive functions and relate these to clinical disease parameters.

**Methods and findings:**

Forty paediatric MS patients, which presented at the German centre for MS in childhood and adolescence, were assessed for cognitive deficits applying a very comprehensive battery of cognitive tests including the Wechsler Intelligence scale and subtests of the D-KEFS for executive functions. The performance of MS patients was compared with a group of age and sex matched healthy controls using between-subjects ANOVAs. Paediatric MS patients performed worse in tests assessing verbal comprehension and fluency, processing speed, memory, calculation skills and other executive functions. Arranged by the cognitive domain, group differences were most pronounced regarding verbal comprehension and fluency for the WISC subtests Comprehension (p = 0.000), Vocabulary (p = 0.003) and Information (p = 0.005); regarding processing speed for the written SDMT (p = 0.001) and the WISC subtest Coding (p = 0.005); regarding memory for the VLMT training (p = 0.007) and the BASIC MLT pattern learning training (p = 0.009); regarding executive functions including working memory for the WISC subtest Arithmetics (p = 0.002), the D-KEFS Design Fluency (p = 0.003) and the Corsi block tapping backward task (p = 0.003). Fluid reasoning was largely intact. Relations of cognitive performance and clinical parameters were assessed in MS patients. Disease duration was associated with a reduced performance in tests belonging to the domains verbal comprehension and fluency (WISC Vocabulary: p = 0.034, WISC Information: p = 0.015) and fluid reasoning (WISC Picture Completion: p = 0.003) as well as the WISC Working Memory Index (p = 0.047). Patients with a disease onset between 11 and 14 years performed better in fluid reasoning (WISC matrix reasoning: p = 0.024) than patients with a disease onset at an age above 14. The number of relapses negatively influenced the visual spatial memory performance (BASIC MLT pattern learning training: p = 0.009).

**Conclusions:**

The distribution of cognitive deficits in a representative group German of paediatric MS patients was similar to the pattern known from other European and North-American cohorts. Paediatric MS patients do have cognitive deficits in executive functions and key qualities necessary for successful school performance. Disease duration, age of onset and the number of relapses influence cognitive performance. Cognitive screenings should be implemented on a regular basis for paediatric MS patients, enabling early intervention.

## Introduction

In 3 to 5 percent of patients afflicted with multiple sclerosis (MS) symptoms start during childhood or adolescence [1, 2]. The inflammatory and neurodegenerative impact of MS meets the brain during a critical time period and may disturb ongoing myelination and other cerebral maturation processes [3]. Whereas permanent physical handicaps are usually rare and mild in paediatric MS (paedMS) patients [4, 5], approximately one third of children and adolescents experience cognitive decline early after onset of disease [6].

Comprehensive reviews have been published recently, summarizing the available studies on cognitive deficits in paedMS patients [7–10]. Similar to adult-onset cases of MS, attention and information processing are frequently impaired [11–13]. Particularly the Symbol Digit Modalities Test (SDMT), an established screening test in adults with MS assessing information processing speed, has been proposed as cognitive screen also in paedMS [14]. Several studies report on deficits in spatial and verbal memory tasks in paedMS patients, including working memory, short- and long-term capacities with variable extent [11, 12, 15, 16]. Impaired language skills are also often noticed in paedMS and may involve receptive and expressive language, particularly detected with complex and speed-dependent linguistic tasks [11–13]. The extent of linguistic impairment appears to be the main neuropsychological difference between children and adults with MS. Language dysfunction is relatively rare for adult patients.

The detection of potential deficits in executive functions and fluid reasoning has not been at the centre of interest of most published studies on cognitive impairment in paedMS. However, cognitive processes such as attentional control, working memory, inhibitory control, flexibility, problem solving and reasoning are important requirements for the achievement of milestones in adolescence including successful school performance, emancipation from the parental home and development towards self-dependence. Therefore, assessing deficits in executive functions and fluid reasoning is of particular interest for paedMS patients. A study by Till and colleagues [17] explicitly focussed on detecting deficits in executive functions in a group of 34 adolescents with paedMS. They found that 44% of patients were impaired in the Trail Making Test (TMT) Part B, which assesses cognitive flexibility, multitasking and simultaneous processing. The TMT Part B is the most frequently applied test in studies assessing executive functions in paedMS. Results are inconsistent, however, with some studies reporting deficits [11, 12, 15, 18] while others do not [19, 20]. Further tests of executive functions, which have occasionally been applied, are the Tower of London Test [19, 21], the Wisconsin Card Sorting Test [13, 17] or its short version [11, 12] and tests resembling the stroop task [16, 17] all with largely inconspicuous results. However, previous studies have found that paedMS patients seem to be particularly vulnerable for deficits in calculation skills [11, 22].

The goal of the present study was to assess the extent and domains of cognitive impairment with a focus on executive functions and fluid reasoning in a German paedMS patient cohort and relate outcomes to clinical disease parameters. Since the available studies in this field have been accomplished in countries with comparable sociodemographic, educational and medical resources, we expected a similar distribution of cognitive impairment in our MS patients as previously imported. Reciprocally, this would allow for an inference for our results regarding executive functions to other cohorts of paediatric MS patients, which have not been investigated accordingly before.

## Materials and methods

### Subjects

Forty German MS patients between 11 and 18 years of age were recruited at the German centre of MS in childhood and adolescence in Göttingen consecutively within a time period of two years, when they presented for their semi-annual check-up. Its catchment area covers all of Germany. All patients met diagnostic criteria of MS according to Krupp et al. [23]; other neurological diseases had been excluded. Additional exclusion criteria were psychiatric diagnoses, severe psychological problems, learning difficulties, insufficient German proficiency and an IQ below 70. We did not include patients younger than 11 years because several of the psychological tests applied in our battery were not applicable to younger subjects. At the time of psychological and clinical evaluation all subjects were both relapse free and not on steroids for at least four weeks. Age and sex matched healthy controls were recruited via advertisements at local schools and in the hospital. Additional to the exclusion of all neurologic illnesses, exclusion criteria for controls were the same as for patients. Written informed consent was obtained from participants and their parents. The ethics committee of the University Medical Center Göttingen approved the study.

### Clinical and neuropsychological assessment

Each patient was seen by a paediatric neurologist for an assessment of clinical history and treatment. A neurologic examination was performed including the Expanded Disability Status Scale (EDSS). A trained psychologist conducted a comprehensive neuropsychological test battery, which required two sessions on separate days, each lasting 90 to 120 minutes. For the detailed description of all tests and their cognitive domains see Table 1.

**Table 1:** Summary of all tests applied in the study.

Domain and Test	Abbr.	Cognitive Function	Short Description
Verbal comprehension and fluency			
WISC-IV / WAIS-IV [25, 26]: Comprehension	WISC CO	Verbal concept formation / reasoning / judgement, knowledge of social conventions and meaning	S answers questions based on his / her understanding of general principles and social situations
WISC-IV / WAIS-IV: Vocabulary	WISC VC	Word knowledge, verbal concept formation, general knowledge	Picture items: S names pictures displaced in the Stimulus Book; Verbal items: S gives definitions for words which are read aloud
WISC-IV / WAIS-IV: Information	WISC IN	General information acquired from culture	S answers questions addressing a broad range of general knowledge topics
WISC-IV / WAIS-IV: Similarities	WISC SI	Verbal concept formation / reasoning, general knowledge, abstract thinking	S is presented with two words that represent common objects / concepts and describes how they are similar
Regensburger Word Fluency Test [27]	RWT	Word fluency, processing speed	S is instructed to first name as many “s”-words, then as many animals as possible in a certain time
K-NEK: Word search [28]	K-NEK WS	Verbal / thinking flexibility, word finding, vocabulary, problem solving	S has to discover “hidden” letters / words interpreting clues such as samples of words with four letters within a limited time
Processing Speed			
Symbol Digit Modality Test [29]	SDMT	Processing speed	S pairs specific numbers with geometric figures using a reference key in a certain time; two trials: written and oral responses
WISC-IV / WAIS-IV: Coding	WISC CD	Processing speed	S copies symbols that are paired with numbers using a key in a certain time limit
d2-R [30]	d2-R	Attention	Rows with the letters “d” and “p” each tagged with 1 to 4 dashes are presented; S has to cross as many “d”s with 2 dashes as possible in a certain time
WISC-IV / WAIS-IV: Symbol Search	WISC SS	Processing speed	S scans a search group and indicates whether the target symbol(s) matches any of the symbols in the search group within a specified time limit
WISC-IV / WAIS-IV: Cancellation	WISC CA	Processing speed	S scans a random and a structured arrangement of pictures and marks target pictures within a specified time limit
Memory (excluding Working Memory)			
Verbal Learning and Memory Test [31]: training	VLMT training	Verbal short-term memory	A list of 15 words is read to S 5 times with an immediate recall each time; an interference trial with different 15 words is then presented and recalled, followed by another recall of the initial list
Verbal Learning and Memory Test: delay	VLMT delay	Verbal long-term memory	A delayed recall of the initial list is performed after 30 min
BASIC-MLT [32]: Pattern learning: training	MLT PL training	Visual-spatial short-term memory	Geometric shapes are presented to S 5 times with a recall each time; an interference list is then presented and recalled, followed by another recall of the initial list
BASIC-MLT: Pattern learning: delay	MLT PL delay	Visual-spatial long-term memory	A delayed recall of the initial list is performed after 30 min
BASIC-MLT: Spatial positioning	MLT SP	Visual-spatial short-term memory	Geometric shapes placed in a 3x3 or 4x4 grate are shown to the subject for 5 seconds one at a time; S has to rebuild the pattern immediately after the template is hidden
Working Memory and Executive Functions			
WISC-IV / WAIS-IV: Arithmetic	WISC AR	Quantitative reasoning, concentration, mental manipulation, calculation skills, working memory	S mentally solves a series of orally presented arithmetic problems within a specified time limit
D-KEFS [33]: Design Fluency	D-KEFS DF	Problem solving, fluency in generating visual patterns, nonverbal creativity, cognitive shifting, inhibition	Condition 3: S is presented a paper with multiple arrays of dots and asked to make as many different designs with the dots as possible using four lines, required to switch between filled and empty dots
Corsi Block Tapping Task [34] (forward and backward task)	CBTT	Visual spatial short-term memory, working memory	Forward task: S has to mimic the instructor who taps a sequence of up to nine spatially separated blocks; Backward task: S watches the sequence and repeats it in backward order
WISC-IV / WAIS-IV: Letter-Number-Sequencing	WISC LN	Auditory recall / processing, attention, working memory	A sequence of numbers / letters is read aloud and S recalls them in ascending / alphabetical order
D-KEFS: Trail Making Test	D-KEFS TMT	Multitasking, divided attention, flexibility of thinking, processing speed	S is instructed to connect a sequence of targets on a sheet of paper as quickly / accurately as possible; we focussed on number-letter switching (Condition 4)
D-KEFS: Colour Word Interference	D-KEFS CWI	Naming and reading speed, verbal inhibition, cognitive flexibility	Condition 4: S sees a page with written colour names, printed in a different-coloured ink and has to name the colour of the ink that the letters are printed in and not read the word; some words are presented inside a box, here, S should read the word and not name the ink colour
WISC-IV / WAIS-IV: Digit Span	WISC DS	Auditory processing, encoding, working memory	Forward: S repeats numbers in the same order as presented aloud; Backward: S repeats numbers in reverse order
Fluid Reasoning			
WISC-IV / WAIS-IV: Matrix reasoning	WISC MR	Nonverbal abstract problem solving, inductive reasoning	S looks at an incomplete matrix and selects the missing portion from five response options
WAIS-IV: Visual Puzzles	WAIS VP	Visual spatial reasoning	S views a completed puzzle and selects three response options that, when combined, reconstruct the puzzle in a limited time
WISC-IV: Picture concepts	WISC PCon	Fluid reasoning, categorization	S is presented with two or three rows of pictures and chooses one picture from each row to form a group with a common characteristic
WAIS-IV: Figure weights	WISC FW	Quantitative / analogical reasoning	S views a scale with missing weight(s) and selects the response option that keeps the scale balanced
D-KEFS: Tower Test	D-KEFS TT	Spatial planning, rule learning, inhibition, establishing and maintaining cognitive set	Five disks that vary in size are presented on a board with three pegs in a predetermined arrangement, the “ending position”; S is asked to move the disks to reproduce that position respecting certain rules
WISC-IV / WAIS-IV: Block design	WISC BD	Visual spatial processing / problem solving /motor construction	Viewing a constructed model or a picture in the Stimulus Book, S uses red-and-white blocks to recreate the designs within a specified time limit
WISC-IV: Picture Completion	WISC PCom	Attention / alertness to visual detail, visual discrimination	S views a picture and then points to or names the important part missing within a specified time limit

Abbreviations not explained in the Table: Abbr.: Abbreviation; S: Subject; BASIC-MLT: Battery for Assessment in Children–Memory and Retentiveness Test; D-KEFS: Delis-Kaplan Executive Function System; K-NEK: Kaufman—Neuropsychological Shortest; WAIS: Wechsler Adult Intelligence Scale Fourth Edition; WISC: Wechsler Intelligence Scale for Children®—Fourth Edition.

### Statistical analysis

All neuropsychological tests were analysed according to the manual. Whenever possible scaled scores were computed, which take age and gender into account. Otherwise, raw scores were used. Note that age and gender were still controlled for as patients and controls were matched accordingly. For each domain and neuropsychological test we tested for statistical differences between patients and controls using between-subjects ANOVAs. A significance level of 5% was adopted. We refrained from computing MANOVAs, because different tests subsumed under the same domain of cognitive functioning may tap into different aspects. As effect sizes we computed standardized mean differences (Cohen’s d) [24]. Effects are considered small if d > = 0.2, medium if d > = 0.5, and large if d > = 0.8. A power analysis showed that effects of d > = 0.66 would result in a statistically significant finding with a power of 0.8 given our final sample size of N = 74. Thus, our sample was large enough to discover medium to large effects with high statistical power.

In addition, we analysed whether clinical parameters of the patients predicted cognitive functioning. To do so, we classified patients according to age of onset (up to 14 years of age vs. older), disease duration (up to one year vs. more than one year), number of relapses (one vs. more than one), and EDSS score (0 and 1 vs. 2, 3, and 4). The specific cut-offs for the groups were chosen taking both clinical meaningfulness into account and the number of subjects in each group. This classification was introduced in order to avoid a bias of a classical correlation analysis by outliers. ANOVAs with clinical parameters as factors were computed for all test scores.

## Results

We initially evaluated 40 German paedMS patients and 60 healthy controls. Of the patients, 37 received a disease modifying therapy: 26 interferon beta, 4 glatirameracetate, 4 natalizumab and 3 fingolimod. For each patient, we selected a sex-matched healthy control with the same age (+/- 3 months) at the time of testing. If more than one healthy control equally matched a patient, we took the control subject which was recruited earlier into the study. From the initial sample, 37 patients and 37 age and sex matched controls were included into the final comparative analyses. Three patients had to be excluded because no healthy control was available. The level of secondary school education was equal for patients and controls in our sample. Regarding parental education, 25% of the fathers and 37.5% of the mothers of paedMS patients had a higher educational level (more than 9 years of school) compared to 43.5% of the fathers and 52.2% of the mothers of the healthy controls. For a detailed description of the study sample and clinical parameters of the patients see Table 2.

**Table 2:** Description of the study sample.

	Patients	Controls
Number	37	37
Males/Females	10/27	10/27
Age (in years)	15.5 ± 1.8	15.3 ± 1.7
Age at disease onset (in years)	13.7 ± 2.6 (7–17)	
Disease duration (in months)	22.5 ± 16.9 (4–73)	
Number of relapses	2.7 ± 2.0 (1–12)	
EDSS score	1.1 ± 1.1 (0–3.5)	

Data is depicted as mean ± SD (range); EDSS: Expanded Disability Status Scale

The results of paedMS patients and matched healthy controls are shown in Table 3. P-Values of statistical comparisons between groups and effect sizes (Cohen’s d) are also provided. First we analysed the overall IQ and indices provided by the Wechsler Intelligence Scales for Children (WISC) and Adults (WAIS). According to their age, 38 subjects were assessed with the WAIS and 36 subjects using the WISC. We assigned each WISC/WAIS subtest and all other individual tests to one of five predefined cognitive domains 1) verbal comprehension and fluency, 2) processing speed, 3) memory excluding working memory, 4) working memory and executive functions and 5) fluid reasoning in order to gain a better insight into domain-specific differences between patients and controls. Assignments were based on the primary cognitive domain targeted by each test as described in Table 1.

**Table 3:** Test results in paediatric MS patients and healthy controls.

Domain and Abbreviation	Score	Patients	Controls	P-value	Effect size
Global measures					
Overall IQ	SS	97.1 ± 18.8	108.9 ± 12.9	0.003**^,^^a^	0.73 ± 0.47
Verbal Comprehension Index	SS	95.9 ± 13.0	107.5 ± 14.6	0.001***^,^^a^	0.84 ± 0.48
Processing Speed Index	SS	98.5 ± 16.5	106.5 ± 10.7	0.017*	0.58 ± 0.47
Working Memory Index	SS	100.7 ±14.7	106.5 ± 14.4	0.097	0.40 ± 0.46
Perceptual Reasoning Index	SS	104.2 ±13.9	108.2 ± 11.9	0.187	0.31 ± 0.46
Verbal comprehension and fluency					
WISC CO	SC	8.5 ± 2.7	11.2 ± 2.9	0.000***^,^^a^	0.96 ± 0.48
WISC VC	SC	9.5 ± 3.1	11.7 ± 2.8	0.003**^,^^a^	0.75 ± 0.47
WISC IN	SC	8.8 ± 2.8	10.8 ± 3.1	0.005**^,^^a^	0.68 ± 0.48
WISC SI	SC	9.8 ± 2.5	11.1 ± 2.3	0.017*	0.54 ± 0.47
RWT “s” words	Raw	20.0 ± 6.2	21.4 ± 5.9	0.315	0.23 ± 0.46
RWT “animal” words	Raw	33.6 ± 8.9	36.4 ± 7.7	0.146	0.34 ± 0.46
K-NEK WS	SC	11.2 ± 2.1	11.4 ± 1.6	0.752	0.11 ± 0.46
Processing speed					
SDMT written	T	50.3 ± 9.1	58.9 ± 11.1	0.001***^,^^a^	0.85 ± 0.48
SDMT oral	T	61.9 ± 14.2	70.4 ± 14.9	0.015*	0.58 ± 0.47
WISC CD	SC	9.8 ± 2.7	11.4 ± 1.9	0.005**^,^^a^	0.69 ± 0.47
d2-R processed objects	SS_d2	102.8 ± 9.0	109.3 ± 12.5	0.010**	0.59 ± 0.50
d2-R concentration	SS_d2	104.9 ± 8.6	110.4 ± 11.5	0.018*	0.54 ± 0.50
WISC SS	SC	10.1 ± 2.8	10.9 ± 2.7	0.263	0.29 ± 0.46
WISC CA	SC	11.7 ± 3.8	11.5 ± 3.1	0.850	0.06 ± 0.46
Memory (excluding working memory)					
VLMT training	T	56.5 ± 10.2	62.3 ± 7.6	0.007**^,^^a^	0.65 ± 0.47
VLMT delay	T	53.0 ± 10.5	58.5 ± 7.3	0.011*	0.61 ± 0.47
MLT PL training	T	49.7 ± 10.0	55.7 ± 8.9	0.009**^,^^a^	0.64 ± 0.47
MLT PL delay	T	52.7 ± 10.7	55.2 ± 8.8	0.299	0.26 ± 0.47
MLT SP	T	48.6 ± 13.8	54.0 ± 12.0	0.077	0.42 ± 0.46
Working memory and executive functions					
WISC AR	SC	9.2 ± 2.4	11.0 ± 2.3	0.002**^,^^a^	0.77 ± 0.48
D-KEFS DF Condition 3	SC	9.7 ± 2.5	11.5 ± 2.6	0.003**^,^^a^	0.71 ± 0.48
CBTT backward	Raw	57.1 ± 18.8	70.6 ± 19.0	0.003**^,^^a^	0.70 ± 0.48
CBTT forward	Raw	59.8 ± 21.2	70.0 ± 29.3	0.099	0.40 ± 0.47
WISC LN	SC	10.3 ± 2.5	11.5 ± 2.5	0.030*	0.62 ± 0.47
D-KEFS TMT Condition 4	SC	10.2 ± 2.4	10.9 ± 2.0	0.136	0.33 ± 0.48
D-KEFS CWI Condition 4	SC	10.3 ± 2.4	10.9 ± 2.5	0.360	0.25 ± 0.46
WISC DS	SC	10.8 ± 3.1	11.3 ± 2.8	0.407	0.20 ± 0.46
Fluid reasoning					
WISC MR	SC	10.4 ± 2.8	11.7 ± 2.0	0.031*	0.54 ± 0.47
WAIS VP	SC	10.5 ± 2.7	11.3 ± 2.8	0.410	0.29 ± 0.66
WISC PCon	SC	11.4 ± 2.3	12.1 ± 2.2	0.393	0.31 ± 0.65
WISC FW	SC	10.6 ± 3.2	9.9 ± 2.8	0.498	0.23 ± 0.66
D-KEFS TT	SC	11.8 ± 3.3	10.2 ± 2.6	0.222	0.17 ± 0.49
WISC BD	SC	10.6 ± 3.3	10.4 ± 3.3	0.794	0.06 ± 0.46
WISC PCom	SC	10.3 ± 3.2	10.6 ± 2.5	0.612	0.11 ± 0.46

For a description of the tests and an explanation of abbreviations see Table 1. Depicted values are mean ± SD. The p-value refers to group differences tested by ANOVA

*: p-value ≤ 0.05

**: p-value ≤ 0.01

***: p-value ≤ 0.001

^a^: significant if number of statistical tests within the domain is taken into account. The effect size is Cohen's d ± 95% confidence interval. SS = standard score: mean 100, SD 15; SS_d2 = standard score: mean 100, SD 10; SC = scaled score: mean 10, SD 3; T = T score: mean 50, SD 10.

In the following, for readability, we report mean test scores in brackets for patient (Pat) and Controls (Con) as well as the P-value (p). For standard deviation and effect size please see Table 3. With respect to global measures of intelligence we found that the average overall IQ was lower in paedMS patients (Pat 97.1, Con 108.9, p = 0.003). They also had significantly lower scores in verbal comprehension (Pat 95.9, Con 107.5, p = 0.001) and processing speed (Pat 98.5, Con 106.5, p = 0.017).

With regard to verbal comprehension and fluency, WISC subtests (CO: Pat 8.5, Con 11.2, p = 0.000; VC: Pat 9.5, Con 11.7, p = 0.003; IN: Pat 8.8, Con 10.8, p = 0.005; SI: Pat 9.8, Con 11.1, p = 0.017) showed a clear impairment of patients compared to controls with medium to large effect sizes. These tests assess verbal concept formation and reasoning, general knowledge, knowledge of social conventions and general information acquired from culture. Semantic and phonemic verbal fluency, verbal flexibility and word finding were not afflicted in our sample of paedMS patients (RWT, K-NEK).

Concerning processing speed, the SDMT, the WISC CD and the d2-R yielded a significantly lower performance in patients (SDMT written: Pat 50.3, Con 58.9, p = 0.001; SDMT oral: 61.9, Con 70.4, p = 0.015; WISC CD: Pat 9.8, Con 11.4, p = 0.005; d2-R processed objects: Pat 102.8, Con 109.3, p = 0.010; d2-R concentration: Pat 104.9, Con 110.4, p = 0.018). Results of subtests from the WISC (SS and CA), which are part of the processing speed index, were not different between the groups. The SDMT written and WISC CD were the tests with the highest effect size in this domain. Both test have a very similar task structure.

In the domain of memory excluding working memory, verbal short- and long-term memory were impaired in paedMS patients (VLMT training: Pat 56.5, Con 62.3, p = 0.007; VLMT delay: Pat 53.0, Con 58.5, p = 0.011). Visual-spatial short-term memory was negatively affected in a demanding task (MLT PL training: Pat 49.7, Con 55.7, p = 0.009) but this was not so clean in an easier task (MLT SP: Pat 48.6, Con 54.0, p = 0.077). Visual spatial long-term memory was not significantly affected (MLT PL delay: Pat 52.7, Con 55.2, p = 0.299). Thus, paedMS patients scored inferior to controls in both visual spatial and verbal memory tasks, but the difference was more pronounced for the latter.

Working memory was impaired in patients as well, although not all tests assessing this function yielded significant group differences. Only the more difficult tests such as the CBTT backward task and the WISC LN showed group-differences with moderate effect sizes (CBTT bw: Pat 57.1, Con 70.6, p = 0.003; WISC LN: Pat 10.3, Con 11.5, p = 0.030). Regarding other executive functions, significant differences between the groups were found in the WISC AR (Pat 9.2, Con 11.0, p = 0.002), assessing calculation skills, and the D-KEFS DF (Pat 9.7, Con 11.5, p = 0.003), which requires problem solving skills, non-verbal creativity, fluency in generating visual patterns, cognitive shifting and inhibition. Other classical tests of executive functions such as the D-KEFS TMT and CWI did not yield any group differences. Motor speed and visual scanning assessed by subconditions of the D-KEFS TMT were not affected in paedMS patients (see S1 Table for results of all D-KEFS DF, TMT and CWI subconditions). Fluid reasoning was largely intact in paedMS patients. A significantly worse performance in patients was only observed in the WISC MR (Pat 10.4, Con 11.7, p = 0.031).

**Fig 1:**
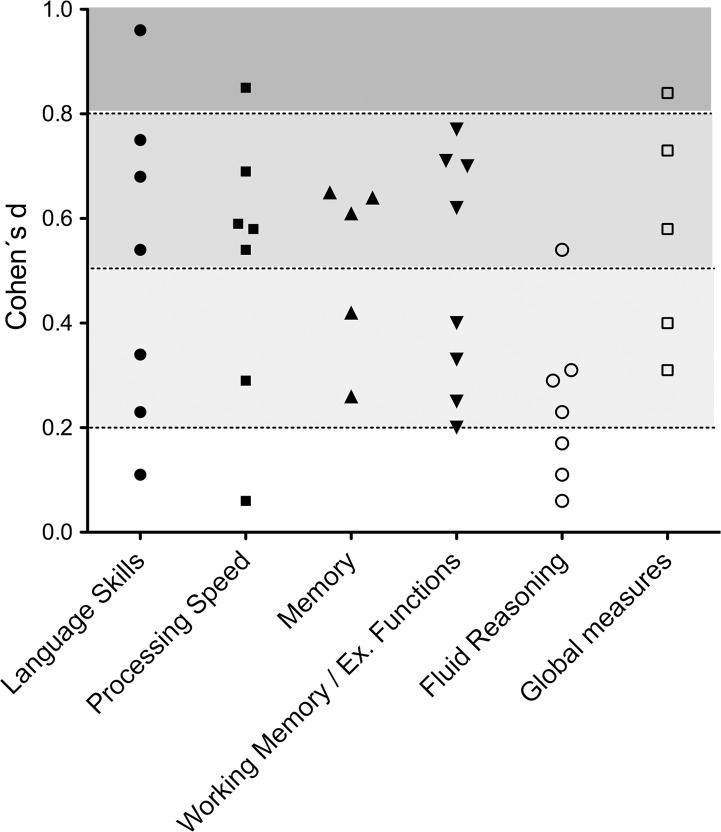
Effect size of all (sub-)tests in each cognitive domain. The underlying shades of grey categorize the effect size: light grey → small effect, grey → moderate effect, dark grey → large effect.

Fig 1 graphically depicts the effect size of all tests applied in our study arranged by cognitive domain. The figure shows that in each cognitive domain except for fluid reasoning small to medium effects were found. Measures with a large effect size were the WISC CO, the SDMT written and WISC verbal comprehension index.

We then analysed whether clinical parameters of paedMS patients were related to cognitive test scores. This analysis is based on all forty patients initially assessed. As explained above, paedMS patients were classified into clinically meaningful groups for age of disease onset, disease duration, number of relapses, and EDSS Score. Findings that were significant or approached significance (p<0.10) are reported in Table 4. Age at disease onset had a significant influence on fluid reasoning (WISC MR: p = 0.024). Interestingly, patients with disease onset at an earlier age showed a consistently better test performance than patients with disease onset at a higher age. As expected, the disease duration negatively affected test performance in several domains (verbal comprehension and fluency: WISC VC: P = 0.034, WISC IN: p = 0.015; fluid reasoning: WISC PCom: p = 0.003; global measures: working memory index: p = 0.047). PaedMS patients with a higher number of relapses performed significantly worse in a spatial short-term memory task (MLT PL training: p = 0.009). The EDSS score did not significantly influence the test performance. The effect sizes for all these observations were medium to large.

**Table 4:** Relations of cognitive measures with clinical disease parameters in paediatric MS patients.

Item	Domain			P-value	Effect size
**Age at disease onset**		Patients 11–14 years old (N = 16)	Patients > 14 years (N = 24)		
Verbal comprehension and fluency	WISC SI	10.4 ± 2.5	9.1 ± 2.4	0.094	0.53 ± 0.64
Fluid reasoning	WISC MR	11.6 ± 2.1	9.6 ± 2.8	0.024*	0.79 ± 0.66
Global measures	Overall IQ	103.4 ± 11.6	92.8 ± 20.6	0.073	0.60 ± 0.65
	Working memory index	105.3 ± 13.8	96.9 ± 14.1	0.076	0.60 ± 0.65
**Disease duration**		Up to one year (N = 14)	Longer than one year (N = 26)		
Verbal comprehension and fluency	WISC VC	10.9 ± 3.0	8.8 ± 2.7	0.034*	0.75 ± 0.67
	WISC IN	10.3 ± 3.7	8.0 ± 1.6	0.015*	0.91 ± 0.68
Processing speed	SDMT written	47.4 ± 10.6	53.0 ± 7.9	0.067	0.63 ± 0.66
Working memory and	WISC LN	11.6 ± 2.6	9.9 ± 2.6	0.056	0.65 ± 0.67
executive functions	WISC DS	11.9 ± 3.3	10.0 ± 2.9	0.068	0.62 ± 0.68
	WISC AR	10.2 ± 2.7	8.8 ± 2.2	0.080	0.59 ± 0.66
Fluid reasoning	WISC PCom	12.3 ± 2.7	9.3 ± 2.7	0.003**	1.11 ± 0.73
Global measures	Verbal comprehension index	100.7 ± 13.9	93.2 ± 11.3	0.078	0.61 ± 0.66
	Working memory index	106.8 ± 16.0	97.1 ± 12.7	0.047*	0.70 ± 0.66
**Number of relapses**		One relapse (N = 9)	More than one relapse (N = 31)		
Verbal comprehension and fluency	WISC CO	7.0 ± 1.5	8.9 ± 2.7	0.066	0.76 ± 0.76
Processing speed	SDMT written	45.9 ± 10.2	52.5 ± 8.6	0.059	0.74 ± 0.76
Memory	MLT PL training	58.4 ± 6.5	47.7 ± 9.8	0.009**	1,16 ± 0.78
Working memory and executive functions	D-KEFS DF	11.4 ± 2.6	9.3 2.5	0.055	0.83 ± 0.85
**EDSS**		Score ≤ 1 (N = 24)	Score > 1 (N = 16)		
Working memory and executive functions	WISC DS	9.9 ± 2.7	11.8 ± 3.4	0.062	0.63 ± 0.65
Processing speed	d2-R processed objects	106.2 ± 10.9	99.8 ± 7.1	0.072	0.67 ± 0.72
	WISC CA	12.6 ± 2.9	10.3 ± 4.4	0.055	0.64 ± 0.65

Abbreviations are explained in Table 1. Only outcomes with p < 0.10 are reported. Values are depicted as mean ± SD.

*: p-value ≤ 0.05

**: p-value ≤ 0.01. The effect size is shown as Cohen's d ± the 95% confidence interval. Only age-adjusted test values were used for correlation with clinical disease parameter which excluded the RWT and CBTT from the analysis.

In order to determine whether the better cognitive performance of patients with an earlier disease onset was due to a shorter disease duration, we also compared the disease duration in the two groups. But contrariwise it turned out that patients with a disease onset at the age of 11–14 years actually had a significantly longer disease duration than patients with disease onset above 14 years (Pat 11–14: 31.8 months, SD 21.0; Pat > 14: 14.9 months (SD 7.6); p = 0.001).

Last, we explored whether there were differences in cognitive impairment between patients receiving basic therapy (interferon beta, glatirameracetate) or no therapy (N = 33 in total) and patients receiving escalation therapy (natalizumab, fingolimod; N = 7). For results see Supplementary Table 1. Although mean values tended to be lower in the escalation therapy group, statistically significant group differences were only observed for few scores (WISC VC: p = 0.013; WISC AR: p = 0.024; verbal comprehension index: p = 0.032). This lack of significant differences is not surprising given the unequal group sizes.

## Discussion

This is the first study assessing cognitive impairment in paedMS patients in the German-speaking countries. We applied a very comprehensive test battery with individual tests chosen based on previously published recommendations for cognitive assessments in MS patients and with a focus on the assessment of executive functions and fluid reasoning. Our results confirm and extend previous findings on cognitive impairment in paedMS patients [7–10]. In the study, we matched each paedMS patient with an age and gender equivalent healthy control. This enabled us to conduct statistical analyses controlling for age and gender even when there were no standard values for a test available. We refrained from introducing a classification of cognitive impairment based on z-scores. Instead, we report significant group differences as important indicator for deficits in paedMS patients.

In our sample, we found a reduced performance of paedMS patients in tests assessing verbal comprehension and fluency, processing speed, memory excluding working memory, working memory and executive functions and fluid reasoning. However, there were important differences concerning the extent of impairment in the different cognitive domains, shown by the number of tests with statistically significant differences between patients and controls in each domain as well as the variable effect size of the individual tests.

A particular focus of our study were deficits in executive functions and fluid reasoning in paedMS patients, since previous studies have only marginally touched this issue. Therefore, we conducted several subtests of the Delis-Kaplan Executive Function System (D-KEFS), which comprises a number of well-designed tests covering key components of verbal and spatial executive function modalities. To our surprise, results showed only deficits in problem solving, nonverbal creativity and cognitive shifting skills (as assessed by the D-KEFS-DF). To our knowledge no other study has ever used the D-KEFS DF in paedMS patients. Several previous studies report an impairment of paedMS patients in simultaneous processing and divided attention (TMT Part B) [30–32] but others show inconsistent results similar to our findings [12, 33]. One other study assessed spatial planning and rule learning (D-KEFS TT) [12], respectively verbal inhibition and cognitive flexibility (D-KEFS CWI) [32], each without finding group differences in accordance with our results. We also found deficits of paedMS patients in working memory and calculation skills (as assessed by the WISC AR). This is in line with previous studies, which found that paedMS patients are especially vulnerable to calculation deficits [11,22]. Based on our findings in the executive function domain, we suggest to use the D-KEFS-DF for future patient assessments of executive function deficits. In contrast to “classical” tests as the stroop task or the Tower of London applied in previous studies the D-KEFS-DF proofed to be more reliable to reveal potential impairments in executive functions.

Working memory, also an executive function, was impaired in paedMS patients but only the more cognitively demanding tests assessing this function (CBTT backward task, WISC LN) yielded significant group differences. This may indicate that basic working memory capabilities remain preserved in paedMS patients (see WISC DS). However, the US study group also applied with WISC DS in their test battery and found a reduced test performance in paedMS patients [16]. Still, more demanding tests may be better suited to assess potential working memory deficits.

We found important group differences in the domain verbal comprehension and fluency, with a clear impairment of paedMS patients compared to controls. PaedMS patients had less deficits in verbal fluency than in verbal comprehension. Pronounced language deficits and deterioration over time have been reported in longitudinal observation study in Italy, with verbal fluency being initially less affected [12, 19, 21]. A large Canadian study also reported important language deficits [13]. However, in a US cohort, language deficits were less pronounced [16]. Our results are closer to the findings in the European and Canadian study groups. Reviewing the available information on language skills in MS patients, we found that our test selection omitted or only marginally touched several aspects such as receptive, expressive and productive language skills. For a sound evaluation of the extent of deficits in language skills in paedMS patients, additional studies are necessary with a focus on this aspect.

Note that several tests in the language domain also require executive function skills (e.g. WISC CO, VC and SI). The high impairment rates of paedMS patients in these tests indicate that executive functions in language may be impaired in patients, which may have an important impact on school performance and communication skills.

Processing speed was also very affected in paedMS patients. This is a frequent finding in studies assessing cognitive impairment in paedMS patients. Deficits were most pronounced in tests that required a translation of numbers into geometric shapes according to a given key (SDMT and WISC CD). Apparently, this type of test is ideally suited to assess processing speed deficits in paedMS patients and is superior to differently designed tests. Our findings also support the recommendation that the SDMT should be used as a screening test for cognitive deficits in MS [14]. Interestingly, the written SDMT yielded a higher effect size than its oral version, probably because it additionally requires visuomotoric skills.

PaedMS patients scored inferior to controls in memory tasks and group differences were larger for verbal memory compared to visual spatial tasks. Possibly, the verbal memory performance was influenced by the frequent impairment of paedMS patients in verbal comprehension. However, in a Canadian study, verbal memory was largely intact although patients had impaired verbal comprehension and fluency [13]. The Italian study group reported a frequent and equal distribution of verbal and spatial memory impairment in paedMS patients [12, 19, 21]. The US network focussed on the assessment of verbal learning and found important deficits in this domain [16]. Thus, memory is consistently impaired in paedMS patients but the deficits regarding verbal and spatial aspects may differ depending on the study population and the administered tests.

Clinical disease parameters turned out to be only modestly associated with some aspects of cognitive performance. The findings give some support to the hypothesis that a longer disease duration goes along with a higher extent of cognitive impairment. No significant relations were found regarding EDSS and the number of relapses. Interestingly, disease onset turned out to be important. PaedMS patients diagnosed at age 14 or younger had better cognitive scores in several domains than patients being diagnosed later. This statistical relation was not biased by disease duration, in fact, patients with a younger disease onset had a longer disease duration than patients with a later disease onset. Our finding is in contrast to a previous study which reports that younger age at disease onset is a positive predictor of cognitive impairment [12]. However, Suppeij and Cainelli reanalysed the data and found that associations of more severe cognitive dysfunction with earlier disease onset largely disappeared after controlling for disease duration [8]. Our own personal experience from daily clinical practise supports the assumption that young patients may be better able to compensate for cognitive impairment, possibly due to the ongoing maturation processes in the central nervous system, which facilitates brain plasticity and restructuring [35].

A limitation of the study is the sample size. Our sample size provided sufficient statistical power to detect moderate to large effects. Nevertheless, to ensure that even small effects will be identified with statistically significant differences more patients and controls are needed. A second potential limitation is that the healthy controls had above average IQ values. Most comparable studies assessing cognitive deficits in paedMS [11–13] have the same issue. We matched patients and controls for the statistical analyses according to gender and age and deliberately decided against including IQ because IQ is likely to be affected by MS and is one feature of cognitive decline. The level of secondary school education was equal for patients and controls in our sample even though the parental education was higher in the control group. We assume that the lower IQ in our paedMS patients is an indicator for their cognitive impairment. However, higher baseline abilities in the control group due to a selection bias cannot be excluded.

## Conclusion

In conclusion, this study confirms the high prevalence of cognitive deficits in paedMS patients with reduced performance particularly in tasks assessing processing speed, verbal comprehension, memory and executive functions. Our results are largely comparable to previous European and North American studies in paedMS patients. Our thorough assessment of executive functions shows that impairments occur in paedMS patients, in particular with respect to design fluency, working memory, calculation skills and verbal concept formation. A longer disease duration and later disease onset go along with more severe cognitive deficits. The frequent impairment of verbal comprehension and calculation skills imposes an important risk factor for deterioration of school performance. In addition to the widely applied cognitive tests, we encourage a systematic inclusion of the D-KEFS DF and the WISC AR for the evaluation of executive functions including calculation skills. Finally, the results imply that all paedMS patients should have access to a comprehensive cognitive screening including verbal and mathematical skills in order to soon provide specific support or disability compensation where this is necessary. Further studies are necessary to give a sound insight into the extent of impairment of receptive and expressive language skills in paedMS patients.

## Supporting information

S1 Table Results of subconditions of D-KEFS tests applied in the study. (DOCX)

S2 Table Comparison of test results in paediatric MS patients receiving escalation therapy (natalizumab, fingolimod) and patients with basic therapy (interferon, glatirameracetate) or without therapy. (DOCX)
