# Supplementary material for: Cognitive deficits including executive functioning in relation to clinical parameters in paediatric MS patients
Source: PLoS One. 2018 Mar 22;13(3):e0194873. doi: 10.1371/journal.pone.0194873 (PMC5864068; doi:10.1371/journal.pone.0194873)
Supplement: S1 Table — (DOCX) [file pone.0194873.s001.docx]

**S1 Table. Results of subconditions of D-KEFS tests applied in the study.**

| **Test** | **Condition** | **Score** | **Patients** | **Controls** | **P-value** | **Effect size** |
| --- | --- | --- | --- | --- | --- | --- |
| Design Fluency | 1: Filled dots | SC | 10.6 ± 2.4 | 11.5 ± 2.6 | 0.111 | 0.36 ± 0.47 |
|  | 2: Empty dots only | SC | 10.4 ± 2.7 | 12.1 ± 2.5 | 0.005^**^ | 0.65 ± 0.47 |
|  | 3: Switching | SC | 9.7 ± 2.5 | 11.5 ± 2.6 | 0.003^**^ | 0.71 ± 0.48 |
| Trail Making Test | 1: Visual scanning | SC | 11.0 ± 2.8 | 11.8 ± 2.4 | 0.202 | 0.31 ± 0.48 |
|  | 2: Number sequencing | SC | 10.2 ± 2.8 | 11.0 ± 2.2 | 0.196 | 0.32 ± 0.48 |
|  | 3: Letter sequencing | SC | 9.9 ± 3.0 | 10.6 ± 2.8 | 0.299 | 0.24 ± 0.48 |
|  | - 4: Number-letter switching | SC | 10.2 ± 2.4 | 10.9 ± 2.0 | 0.136 | 0.33 ± 0.48 |
|  | 5: Motor speed | SC | 10.4 ± 2.3 | 10.6 ± 2.1 | 0.686 | 0.09 ± 0.48 |
| - Colour Word Interference | - 1: Colour naming | SC | 8.9 ± 2.9 | 10.0 ± 2.1 | 0.066 | 0.43 ± 0.47 |
|  | - 2: Word reading | SC | 10.0 ± 2.4 | 9.7 ± 4.1 | 0.745 | 0.09 ± 0.46 |
|  | - 3: Inhibition colour naming | SC | 10.5 ± 2.4 | 9.5 ± 4.5 | 0.208 | 0.28 ± 0.46 |
|  | - 4: Inhibition switching | SC | 10.3 ± 2.4 | 10.9 ± 2.5 | 0.360 | 0.25 ± 0.46 |

For a description of the tests see Table 1. Depicted values are mean ± SD. The p-value refers to group differences tested by ANOVA: ^*^: p-value ≤ 0.05, ^**^: p-value ≤ 0.01, ^***^: p-value ≤ 0.001. The effect size is Cohen´s d ± 95% confidence interval. SC = scaled score: mean 10, SD 3.
