# Supplementary material for: Cognitive deficits including executive functioning in relation to clinical parameters in paediatric MS patients
Source: PLoS One. 2018 Mar 22;13(3):e0194873. doi: 10.1371/journal.pone.0194873 (PMC5864068; doi:10.1371/journal.pone.0194873)
Supplement: S2 Table — (DOCX) [file pone.0194873.s002.docx]

**S2 Table.** **Comparison of test results in paediatric MS patients receiving escalation therapy (natalizumab, fingolimod) and patients with basic therapy (interferon, glatirameracetate) or without therapy.**

| **Domain** | **Abbreviation** | **Score** | **Escalation therapy (N=7)** | **Basic or no therapy (N=33)** | **P-value** | **Effect size** |
| --- | --- | --- | --- | --- | --- | --- |
| Global measures | Overall IQ | SS | 93.6 ± 10.7 | 99.1 ± 16.2 | 0.474 | 0.31 ± 0.82 |
|  | Verbal Comprehension Index | SS | 86.3 ± 11.9 | 97.6 ± 12.1 | 0.032^*^ | 0.93 ± 0.85 |
|  | Processing Speed Index | SS | 100.9 ± 4.8 | 104.4 ± 14.9 | 0.544 | 0.26 ± 0.82 |
|  | Working Memory Index | SS | 92.0 ± 10.9 | 102.8 ± 14.4 | 0.070 | 0.78 ± 0.84 |
|  | Perceptual Reasoning Index | SS | 99.1 ± 17.7 | 99.1 ± 16.2 | 0.991 | 0.00 |
| Language skills | WISC CO | SC | 7.1 ± 2.7 | 8.7 ± 2.6 | 0.147 | 0.61 ± 0.83 |
|  | WISC VC | SC | 7.0 ± 2.1 | 10.1 ± 2.9 | 0.013^*^ | 1.11 ± 0.86 |
|  | WISC IN | SC | 7.9 ± 2.0 | 8.9 ± 2.8 | 0.348 | 0.37 ± 0.83 |
|  | - WISC SI | - SC | 8.3 ± 2.6 | 9.9 ± 2.4 | 0.117 | 0.66 ± 0.83 |
|  | RWT “s” words | - Raw | 18.9 ± 10.5 | 20.5 ± 5.5 | 0.554 | 0.24 ± 0.82 |
|  | RWT “animal” words | - Raw | 28.9 ± 13.5 | 34.4 ± 7.7 | 0.147 | 0.61 ± 0.83 |
|  | - K-NEK WS | - SC | 10.3 ± 2.3 | 11.4 ± 2.1 | 0.227 | 0.51 ± 0.83 |
| - Processing speed | SDMT written | - T | 52.7 ± 9.5 | 50.2 ± 9.3 | 0.515 | 0.27 ± 0.82 |
|  | - SDMT oral | - T | 67.6 ± 15.5 | 61.2 ± 14.0 | 0.287 | 0.45 ± 0.83 |
|  | - WISC CD | - SC | 10.3 ± 3.1 | 9.9 ± 2.6 | 0.713 | 0.15 ± 0.82 |
|  | d2-R processed objects | - SS_d2 | 100.1 ± 5.8 | 103.5 ± 9.5 | 0.387 | 0.38 ± 0.85 |
|  | d2-R concentration | - SS_d2 | 101.1 ± 3.4 | 106.0 ± 9.2 | 0.191 | 0.59 ± 0.85 |
|  | - WISC SS | - SC | 9.3 ± 3.8 | 10.4 ± 2.5 | 0.344 | 0.40 ± 0.83 |
|  | - WISC CA | - SC | 11.3 ± 4.4 | 11.7 ± 3.7 | 0.823 | 0.11 ± 0.82 |
| - Memory (excluding working memory) | - VLMT training | - T | 54.8 ± 11.1 | 57.5 ± 10.1 | 0.531 | 0.26 ± 0.82 |
|  | - VLMT delay | - T | 53.2 ± 12.5 | 53.5 ± 10.2 | 0.938 | 0.03 ± 0.82 |
|  | - MLT PL training | - T | 51.1 ± 8.4 | 49.8 ± 10.4 | 0.747 | 0.13 ± 0.82 |
|  | - MLT PL delay | - T | 52.4 ± 8.1 | 53.2 ± 11.3 | 0.859 | 0.07 ± 0.83 |
|  | - MLT SP | T | 40.6 ± 5.2 | 50.5 ± 14.2 | 0.079 | 0.75 ± 0.84 |
| - Working memory and executive functions | - WISC AR | - SC | 7.4 ± 1.7 | 9.7 ± 2.4 | 0.024^*^ | 1.00 ± 0.85 |
|  | - D-KEFS DF Condition 3 | - SC | 8.1 ± 1.6 | 10.0 ± 2.6 | 0.088 | 0.78 ± 0.85 |
|  | CBTT backward | Raw | 52.3 ± 20.5 | 58.4 ± 18.2 | 0.446 | 0.33 ± 0.83 |
|  | CBTT forward | Raw | 61.2 ± 17.1 | 60.4 ± 22.6 | 0.896 | 0.06 ± 0.83 |
|  | WISC LN | SC | 9.6 ± 3.4 | 10.7 ± 2.5 | 0.303 | 0.41 ± 0.83 |
|  | D-KEFS TMT Condition 4 | SC | 9.4 ± 3.6 | 10.4 ± 2.0 | 0.339 | 0.42 ± 0.83 |
|  | D-KEFS CWI Condition 4 | SC | 9.6 ± 3.0 | 10.6 ± 2.2 | 0.302 | 0.43 ± 0.83 |
|  | - WISC DS | - SC | 9.6 ± 2.5 | 11.3 ± 3.1 | 0.258 | 0.47 ± 0.83 |
| - Fluid reasoning | - WISC MR | SC | 9.9 ± 2.5 | 10.6 ± 2.8 | 0.511 | 0.25 ± 0.82 |
|  | - WAIS VP | - SC | 11.5 ± 1.0 | 10.2 ± 2.8 | 0.373 | 0.50 ± 1.10 |
|  | - WISC PCon | - SC | 11.0 ± 2.6 | 11.5 ± 2.3 | 0.741 | 0.21 ± 1.25 |
|  | - WISC FW | - SC | 8.3 ± 3.3 | 11.1 ± 2.8 | 0.094 | 0.97 ± 1.13 |
|  | - D-KEFS TT | - SC | 10.5 ± 2.8 | 10.9 ± 2.0 | 0.465 | 0.59 ± 0.96 |
|  | WISC BD | SC | 9.3 ± 2.1 | 10.7 ± 3.5 | 0.323 | 0.42 ± 0.83 |
|  | WISC PCom | SC | 8.9 ± 3.4 | 10.6 ± 2.9 | 0.181 | 0.57 ± 0.83 |

For explanation of abbreviations see Table 1. Values are depicted as mean ± SD. The p-value refers to group differences: ^*^: p-value ≤ 0.05. The effect size is shown as Cohen´s d ± the 95% confidence interval. SS = standard score: mean 100, SD 15; SS_d2 = standard score: mean 100, SD 10; SC = scaled score: mean 10, SD 3; T = T score: mean 50, SD 10.
